# pH-Dependent Solution Structure and Activity of a Reduced Form of the Host-Defense Peptide Myticin C (Myt C) from the Mussel *Mytilus galloprovincialis*

**DOI:** 10.3390/md11072328

**Published:** 2013-07-04

**Authors:** Alicia Martinez-Lopez, Jose Antonio Encinar, Regla Maria Medina-Gali, Pablo Balseiro, Pablo Garcia-Valtanen, Antonio Figueras, Beatriz Novoa, Amparo Estepa

**Affiliations:** 1 Molecular and Cell Institute, University Miguel Hernández (IBMC-UMH), Elche 03202, Spain; E-Mails: alicia.martinez@umh.es (A.M.-L.); jant.encinar@umh.es (J.A.E.); pablo.garciav@umh.es (P.G.-V.); 2 LABIOFAM Pharmaceutical Laboratories, La Habana 17800, Cuba; E-Mail: reglita2000@yahoo.com; 3 Instituto de Investigaciones Marinas (IIM), CSIC, Vigo 36208, Spain; E-Mails: pablobal@iim.csic.es (P.B.); antoniofigueras@iim.csic.es (A.F.); virus@iim.csic.es (B.N.)

**Keywords:** myticin, mussel, HDPs, AMPs, VHSV, chemotaxis, pH, structure, infrared spectroscopic, liposomes

## Abstract

Myticin C (Myt C) is a highly variable host-defense peptide (HDP) associated to the immune response in the mediterranean mussel (*Mytilus galloprovincialis*), which has shown to be active across species due to its strong antiviral activity against a fish rhabdovirus found in fish cells overexpressing this HDP. However, the potential antimicrobial properties of any synthetic analogue of Myt C has not yet been analysed. Thus, in this work we have synthesised the sequence of the mature peptide of Myt C variant c and analysed the structure activity relationships of its reduced (non-oxidized) form (red-MytCc). In contrast to results previously reported for oxidized isoforms of mussel myticins, red-MytCc was not active against bacteria at physiological pH and showed a moderate antiviral activity against the viral haemorrhagic septicaemia (VHS) rhabdovirus. However, its chemotactic properties remained active. Structure/function studies in neutral and acid environments by means of infrared spectroscopy indicated that the structure of red-MytCc is pH dependent, with acid media increasing its alpha-helical content. Furthermore, red-MytCc was able to efficiently aggregate artificial phospholipid membranes at low pH, as well as to inhibit the *Escherichia coli* growth, suggesting that this activity is attributable to its more structured form in an acidic environment. All together, these results highlight the dynamic and environmentally sensitive behavior of red-Myt C in solution, and provide important insights into Myt C structure/activity relationships and the requirements to exert its antimicrobial/immunomodulatory activities. On the other hand, the pH-dependent direct antimicrobial activity of Myt C suggests that this HDP may be a suitable template for the development of antimicrobial agents that would function selectively in specific pH environments, which are sorely needed in this “antibiotic-resistance era”.

## 1. Introduction

The development of antibiotics has significantly improved the health quality of humans and animals. However, the overuse of antibiotics has prompted the appearance of multi-drug resistant microorganisms. To date, the antibiotic development pipeline is devoid of imminent solutions to this urgent problem. Furthermore, the considerable drop in the development of new classes of antibiotics by pharmaceutical companies is aggravating this issue [1]. On the other hand, conventional antibiotics are not useful against viruses and some of the few antivirals available are also compromised by the generation of resistant virus strains [2].

Due to the scarcity of anti-infective agents to combat both human and veterinary infectious diseases and the growing specter of untreatable infections, it is imperative to find novel anti-infective agents to solve this steadily-evolving health problem. Currently, the therapeutic potential of natural anti-infective agents such as host defense peptides (HDPs) and their possible use as alternative chemical food preservatives or substitutes for antibiotics and fungicides is being seriously considered.

HDPs are gene-encoded components of the immune system that have been selected by evolution as crucial agents of the first line of defense against invading microbes [3,4] in all living organisms.

Overall, HDPs are short cationic peptides that can be constitutively expressed and/or rapidly induced to interact directly with infectious agents and/or modulate immunoreactions involved in the defense against pathogenic microorganisms [3,5,6]. Because (i) their mechanism of direct action, mainly based on their interaction with the lipid cell membranes of pathogens, and (ii) their multiple and simultaneous modes of action, HDP resistance is difficult to develop for the pathogens [2].

In this regard, marine organisms, and specially these belonging to the invertebrate group, represent a valuable and insufficiently explored source of new HDPs. Some marine organisms lack adaptive immune systems (e.g., invertebrates) or have very primitive ones (e.g., fish) and yet they still are continuously challenged by microbes and the aggressive marine environment; this makes evident the fact that they must rely on their innate immune system effector molecules to fight pathogens, more so than other organisms with developed adaptive immune systems such as mammals [7]. In fact, HDPs represent the major invertebrate humoral defense system against infection, and show a diverse spectrum of mechanisms of action, some of them related to plasma membrane disturbance and lethal alteration of microbial integrity [5].

In this context, we have focused our attention in one HDP found in the Mediterranean mussel (*Mytilus galloprovincialis*), the Myt C. Among other mussel HDPs the Myt C is the only one known to be expressed at larval stages, and among the three different isoforms (A, B and C) of mussel myticins described so far, the isoform C is the most expressed transcript in adults [8,9]. Furthermore, it has been shown that Myt C plays a significant role in the molluscan immune response against pathogens and external aggressions and what is more important, recombinant Myt C peptides have strong antiviral activity against fish viruses in fish cell lines [10], suggesting that Myt C is active across species. The high expression of Myt C and the impressive sequence variability of the transcripts have been related with the high resistance of mussels to infections even though it is a filter feeder organism that can inhabit polluted locations and even feed on bacteria [8,11,12,13].

After analysing the direct antimicrobial activity and the properties as innate inmune effector of synthetic reduced MytCc (red-MytCc) as well as its structure/activity relationships we show the dynamic and environmentally sensitive behavior of this type of HDP in solution. Our results depict Myt C as a potential new and versatile marine-peptide template for the develpoment of bioactive products with application in different fields, for example, the protection of aquacultured species—the most important source of human fish/molluscan protein.

Moreover, marine proteins have been reported to be sufficiently immunogenic in mammalian species to promote inflammation and recruit immune enhancers without necessarily generating antibodies [5]; these features open a new door for the use of these HDPs as effective drugs in human and veterinary medicine.

## 2. Results

### 2.1. Antibacterial and Antiviral Activity of Synthetic MytCc at Neutral pH

As a first step towards the characterisation of the direct antimicrobial activity of synthetic MytCc, we evaluated the ability of this HDP to inhibit the growth of *E. coli* in water solution and at neutral pH. Unexpectedly, synthetic red-MytCc was not able to inhibit the *E. coli* growth at any of the concentrations tested (from 0.01 to 1 mg/mL) in these experimental conditions (data not shown).

**Figure 1:**
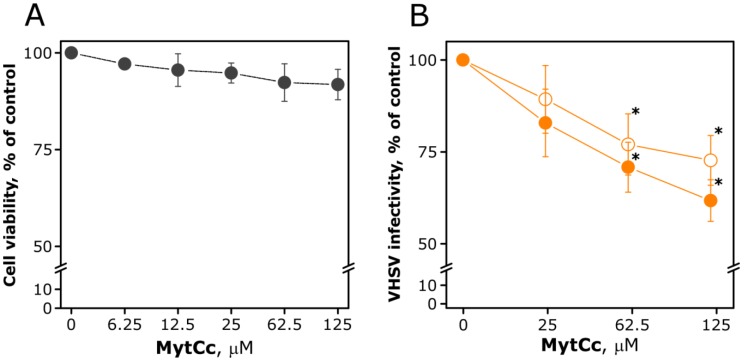
Cytotoxicity (**A**) and antiviral activity (**B**) of synthetic red-MytCc. (**A**) Epithelioma Papulosum Cyprini (EPC) cells were incubated with increased concentrations of synthetic red-MytCc and 72 h post-treatment, the potential cytotoxicity of the peptide evaluated by means of a MTT assay. The data are mean ± SD of two independent experiments, each performed in triplicate. (**B**) EPC cell monolayers, grown in 96-well plates, were infected either with VHSV in the presence of (reduced MyticinC) red-MytCc or with VHSV preincubated with red-MytCc. Twenty-four hours later, VHSV infectivity was estimated by counting the number of foci of VHSV infected cells by the immunostaining focus assay described in Section 4. The data are the mean ± SD from two independent experiments, each performed in triplicate. Symbols: open orange circles, VHSV infectivity when red-MytCc is present during the infection time; solid orange circles, VHSV infectivity after being overnight preincubating with red-MytCc at 14 °C. Asterisks indicate significant differences (*p* *<* 0.05) in viral infectivity relative to control cells.

Next, we wanted to evaluate whether or not the synthetic peptide showed the antiviral activity against the fish rhabdovirus of viral haemorrhagic septicaemia (VHSV) [14] that it exhibited in cells transfected with a construct encoding the cDNA sequence to the mature peptide MytCc [10]. For that, the ability of red-MytCc to reduce VHSV infectivity was studied by (i) pre-incubating increasing concentrations of synthetic red-MytCc with 103 ffu of cell-free VHSV in 25 μL of serum-free cell culture medium and then infecting the cells with the red-MytCc-VHSV mixtures and (ii) adding different concentrations of synthetic red-MytCc at infection time. In both approaches a dose-dependent moderate antiviral activity of red-MytCc could be observed (Figure 1B). Maximal inhibition of viral infectivity (40%) was observed when 125 μM of red-MytCc were pre-incubated with VHSV. Moreover, no significant differences were observed between the treatments.

On the other hand, no cytotoxicity was observed when the Epithelioma Papulosum Cyprini (EPC) cell monolayers were treated with synthetic red-MytCc at concentrations up to 125 μM during 72 h, indicating that the effects of red-MytCc on VHSV infectivity were not due to nonspecific cytotoxicity (Figure 1A).

### 2.2. Chemotactic Properties of Synthetic Red-MytCc at Neutral pH

**Figure 2:**
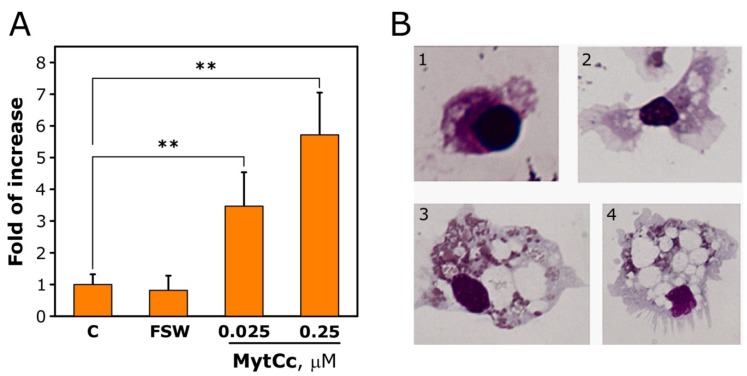
Chemotactic activity of synthetic red-MytCc. Hemocytes from individual mussels were seeded in the upper chamber and synthetic red-MytCc at different concentration in the lower chamber. (**A**) For each mussel the number of migrating cells to the lower chambers was calculated as the mean ± SD of the cellular counting of five different microscopic fields, relative to hemocytes migrating to lower chambers containing FSW. Results are shown as the mean ± SD of 8 individual mussels and expressed as fold of increase of the number of migrating cell related to control (cell migrating in chamber containing red-MytCc in both upper and lower compartments at a concentration of 0.25 μM). Asterisks denote statistically significant differences when comparing with control chambers containing red-MytCc at 0.25 μM in the upper and lower chamber (*p* *<* 0.01). (**B**) 1 and 2, Representative hemocytes after migrating to the lower chamber containing FSW; 2 and 3, representative hemocytes after migrating to the lower chamber containing synthetic red-MytCc at 0.25 μM.

We tested this activity on eight individual mussels and in all of them, synthetic red-MytCc induced an increment of hemocytic chemotaxis compared with controls (Figure 2A), Synthetic red-MytCc at 0.025 and 0.25 μM increased the hemocytes migration by 5- and 3-fold related to controls, respectively. There was no migration in the absence of gradient concentration of the synthetic red-MytCc. During migration, the morphology of hemocytes was altered (Figure 2B). Hemocytes in contact with synthetic red-MytCc were bigger in size, with abundant eosinophilic granules and presence of vacuoles. This change was not apparent in control haemocytes migrating towards FSW. Therefore, synthetic red-MytCc possesses chemotactic properties at neutral pH and low concentration (500-fold lower that the one needed to inhibit VHSV infectivity up to 40%).

### 2.3. pH-Dependence Membrane Destabilization Induced by the Red-MytCc Peptide

**Figure 3:**
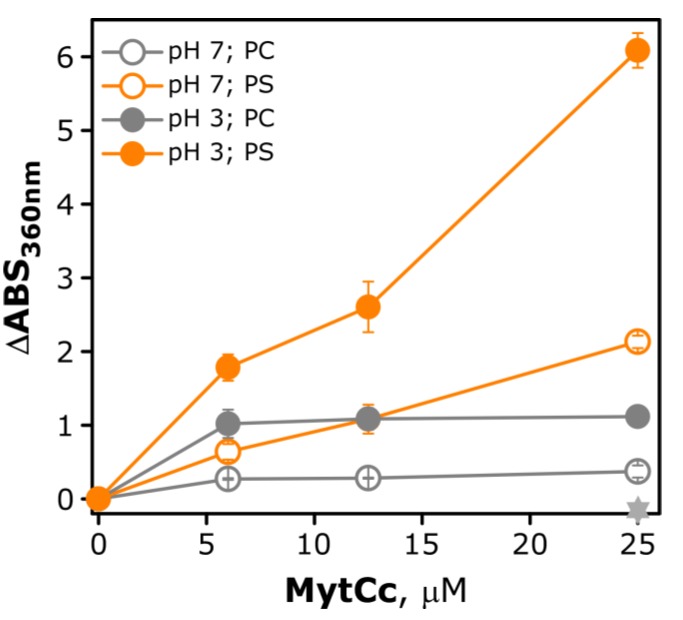
Membrane aggregation induced by synthetic red-MytCc. Changes in the absorbance at 360 nm induced by the interaction of the myticine C peptide with phospholipid vesicles. To 1 mL of vesicles (0.14 mM) of PS or PC in medium buffer at the appropriate pH, aliquots of peptide from a 2.5 mM stock solution in DMSO were added. Symbols: open grey circles, PC at pH = 7.0; open orange circles PS at pH = 7.0; solid grey circles PC at pH = 3.0; solid orange circles PS at pH = 3.0 and start, DMSO alone at the concentration corresponding to the highest Myt C concentration used. The absorbance was measured after incubating the sample for 1 h at 37 °C. The results shown are representative of those obtained for three independent experiments.

Figure 3 shows the changes in the absorbance at 360 nm after mixing PS or PC vesicles with the synthetic red-MytCc at various phospholipid/peptide ratios, and at pH = 3 or pH = 7. Bearing in mind that the lipid or synthetic red-MytCc isolated have no absorbance at 360 nm, the absorbance values that appear when peptide is added to the lipid vesicles should be due to the scattered light produced by the peptide-induced aggregation or increase in size of lipid vesicles. At acidic pH the synthetic red-MytCc interacts with either PS or PC, although to a higher extent than at neutral pH. In the presence of PC the increase in absorbance was almost negligible at neutral pH, and clearly lower than in the presence of PS at acidic and neutral pHs, indicating the specificity of the observed peptide–phospholipid interaction in solution. Major changes in absorbance, caused by the presence of the synthetic red-MytCc (5-fold) were observed at acidic pH and a peptide concentration of 25 μM (approximately 100 μg/mL).

### 2.4. pH-Dependent Secondary Structure of Synthetic Red-MytCc

**Figure 4:**
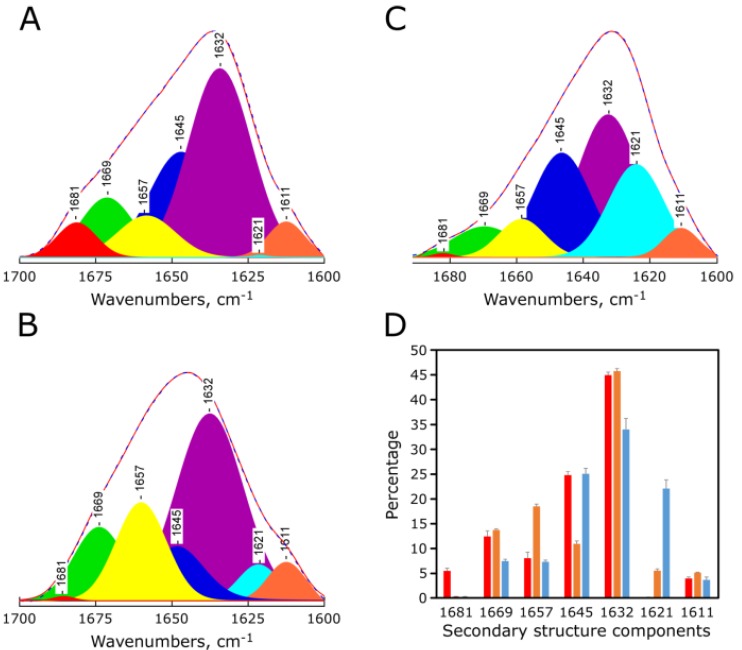
pH-dependent secondary structure of synthetic red-MytCc. Representative amide I′ band profiles in the infrared spectra of synthetic red-MytCc at pH = 7 (panel **A**), synthetic red-MytCc at pH = 3 (panel **B**), and synthetic red-MytCc and PC lipids at pH = 7 (panel **C**). All **A**, **B**, **C** panels include (i) the recorded amide I′ band spectral envelope (continuous line), (ii) the component bands obtained by decomposition of the amide I′ band and (iii) the reconstruction of the amide I′ band from the observed spectral components (dashed line). Band assignments are: 1657 cm^−1^ to α-helix; 1669 and 1681 cm^−1^ to β-turns; 1632 and 1621 cm^−1^ to intramolecular and intermolecular vibrations of β-sheets, respectively; 1645 cm^−1^ to non-ordered conformations, including open loops [19]; 1611 cm^−1^ to tyrosine side chain. Panel (**D**) shows the estimated secondary structural elements (in percentages) for synthetic red-MytCc at pH = 7 (red bars), pH = 3 (orange bars), and synthetic red-MytCc with PC lipids at pH = 7 (blue bars). Results in the latter panels are given as means ± S.E., *n =* 3.

The amide I band (called amide I′ when taken in a D2O medium) comprises the 1600–1700 cm^−1^ infrared spectral region and results primarily from stretching vibrations of C=O groups in peptide bonds [15]. The exact frequencies of such vibrations depend on the nature of the hydrogen bonding involving the C=O groups, which in turn, is determined by the particular secondary structure adopted by the protein [16]. Thus, the amide I′ band contour represents the composition of overlapping spectral components of characteristic frequencies, which are assigned in H_2_O and in D_2_O to different secondary structural motifs in both soluble and membrane-bound proteins [17]. Figure 4 shows infrared amide I′ band of synthetic red-MytCc at pH = 7 (Figure 4A), pH = 3 (Figure 4B), and in the presence of PC lipids (Figure 2C). Quantitative estimates on the protein’s secondary structure were obtained by decomposition of the amide I’ band into its spectral components [18] and confirm that indeed, the changes in synthetic red-MytCc spectral shape between neutral and acidic (alone or in the presence of PC lipid vesicles) pHs samples are primarily due to an increase in the latter of the alpha-helix spectral component at 1657 cm^−1^ (Figure 4D), and a decrease of the random-coil component at 1645 cm^–1^. No changes were observed in the total percentage of β-sheet, although the percentage of intermolecular beta-sheet clearly increases in the presence of PC lipids, indicating a greater aggregation of the synthetic red-MytCc.

**Figure 5:**
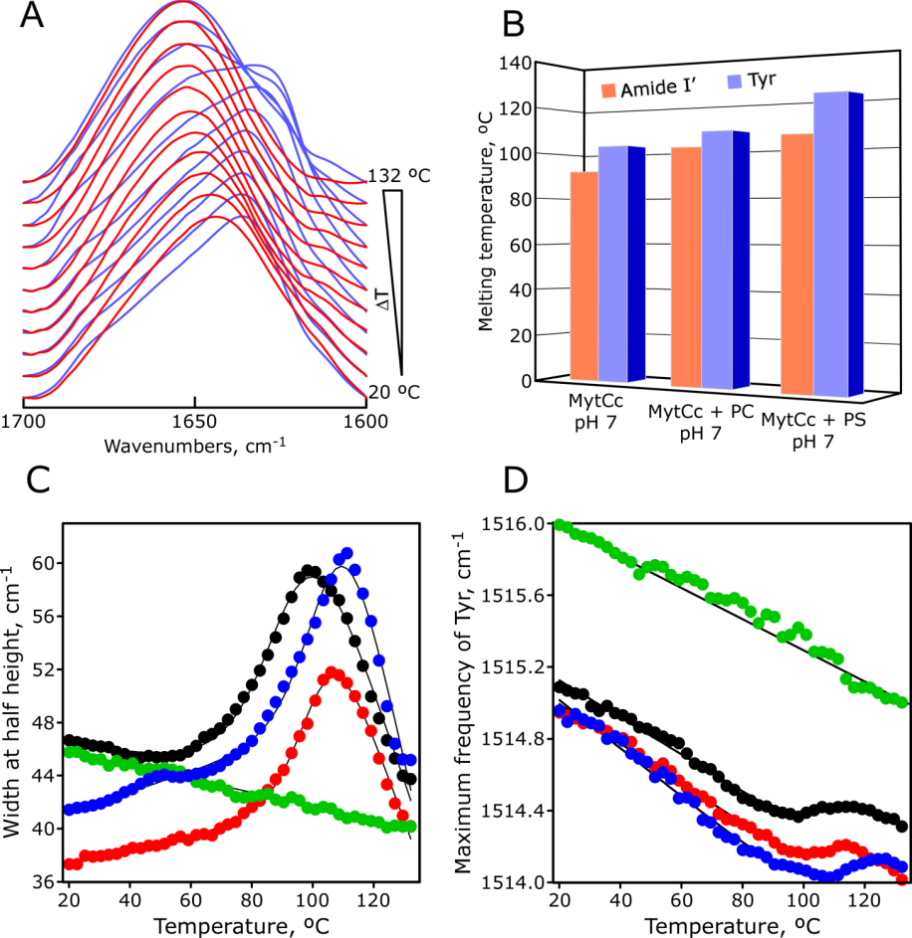
pH-dependent thermal stability of synthetic red-MytCc. Infrared amide I′ band stacked spectra of synthetic red-MytCc at pH = 3.0 (red) and pH = 7.0 (blue) acquired at increased temperatures (panel **A**). Panel **B** shows a bar graph with thermal midpoint, T*_m_*, estimated from the data represented in panel **C** (Amide I′) and panel **D** (Tyr). Panel **C** shows temperature-dependent amide I′ band width at half height for synthetic red-MytCc at pH = 7 (solid black circles), and pH = 3 (solid green circles), synthetic red-MytCc + PS lipid at pH = 7 (solid blue circles); and synthetic red-MytCc + PC lipid at pH = 7 (solid red circles). The solid line represents the best fits of experimental data to a modified sigmoid equation (see Materials and Methods). Panel **D** represents the temperature-dependence of the Tyr band maximum frequency of infrared spectrum for synthetic red-MytCc under different conditions. They have used the same symbols as in panel **C** and also the solid line represents the best fits of experimental data to a modified sigmoid equation.

### 2.5. pH-Dependent Thermal Stability of Synthetic Red-MytCc

Infrared spectroscopy is a powerful method for investigation of secondary and tertiary structures, by following spectroscopic changes of amide I′ and, Tyr (appearing at 1515 cm^–1^) bands, respectively. The thermal dependence of the amide I′ band (Figure 5A,C) has been used to assess the stability of the protein secondary structure. Different plots can be used to characterize the thermal profile, e.g., bandwidth *vs.* temperature or the ratio (absorbance of the most intense component/absorbance of the emerging band) *vs*. temperature, *etc.* [19]. Figure 5C shows the amide I′ band width at half height *vs.* temperature in different conditions. The most striking feature is that the red-MytCc peptide at pH = 3 does not undergo any thermal transition in the temperature range studied. This is consistent with the higher percentage of α-helix described above. In all three conditions, synthetic red-MytCc experience a thermal transition at high temperatures: 92 °C at pH = 7, 101 °C in the presence of PC lipids at pH = 7, and 105 °C in the presence of PS lipids at pH = 7. We note that the higher transition temperatures correspond with higher levels of aggregation lipid induced peptide mentioned above. Thermal unfolding of protein involves not only loss of secondary structure, but also changes in the microenvironment of side-chain groups. Aromatic ring stretching vibrations of tyrosine at 1515 cm^–1^ provide a specific local monitor for tertiary structural changes [20]. Figure 5D shows changes in the maximum frequency of the infrared tyrosine band *vs.* the increase of temperature. In a similar fashion, that for amide I′ band of red-MytCc peptide at pH = 3 does not display any thermal transition as follows the infrared Tyr band (Figure 5D). For the other experimental conditions, the thermal midpoint denaturation estimated from the sigmoid behavior of this tyrosine band is very close to the estimated by amide I′ band (Figure 5B).

**Figure 6:**
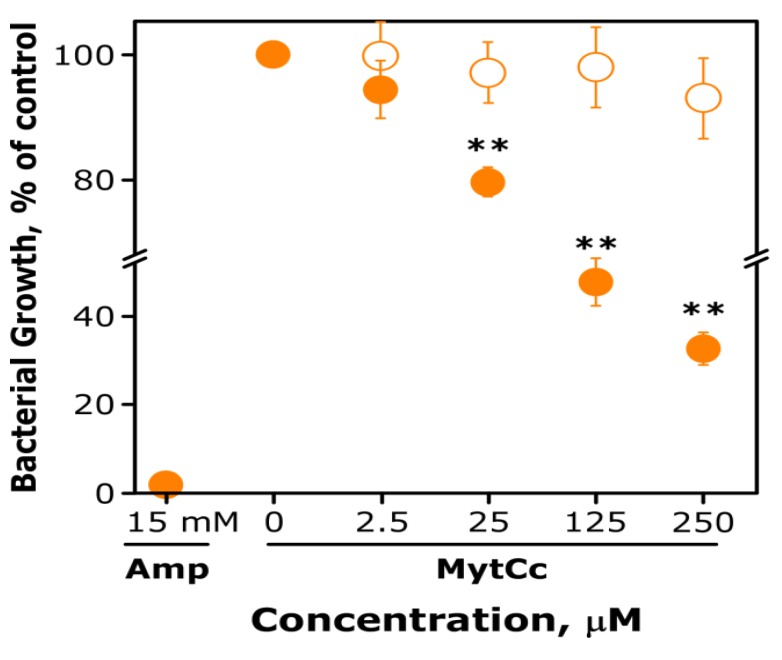
Antibacterial activity of synthetic red-MytCc at low pH. Suspensions of ~10^3^ CFU of *E. coli* in of LB media were grown in the absence or presence of different concentrations of synthetic red-MytCc (from 2.5 to 250 μM) diluted in water at pH 3. After overnight incubation at 37 °C and the *E.coli* growth was determined colorimetrically using iodonitrotetrazolium violet dye (INT). The data are the mean ± SD from two independent experiments, each performed in hexaplicate. Asterisks indicate significant differences (*p* *<* 0.01) in bacterial growth relative to control cultures (bacteria grown in the absence of red-MytCc).

### 2.6. Antibacterial and Antiviral Activity of Synthetic Red-MytCc at Acid pH

Taking into account the results of biophysical assays, we hypothesised that the antibacterial activity of synthetic red-MytCc should be pH-dependent. To evaluate this possibility, *E. coli* suspensions were incubated with different concentrations of red-MytCc diluted in water at pH 3 or 7. Under those conditions, red-MytCc showed bactericidal activity but only when the peptide was added from a stock solution at pH 3 (Figure 6). Maximal inhibition of *E. coli* growth (70%) was observed at 1 mg/mL of synthetic red-MytCc. As expected, ampicillin was able to completely abrogate the *E. coli* growth but at concentrations of 14 mM (Figure 6).

## 3. Discussion

Today, marine environments are considered to be the largest remaining reservoir of natural molecules waiting for the evaluation of their diverse drug activities [21].

MytC is a marine HDP expressed by Mediterranean mussel (*M. galloprovincialis*) hemocytes and belongs to the previously identified Myt peptide family [22] with interesting properties to be used as an enhancer of fish defenses in stressful environments and as a model molecule for improving the design of fish antimicrobial drugs. Moreover, it is known that recombinant MytC can act as an antiviral agent against pathogenic fish viruses as well as a chemokine/cytokine-like molecule [10]. However, the activity of a synthetic peptide encoding the sequence of the mature reduced peptide of any MytC variant has not been evaluated so far. Thus, in this work we have synthesised the sequence of the mature peptide of MytC variant c and analysed the structure activity relationships of its reduced (non-oxidized) form, (red-MytCc). To carry out this study we chose a reduced form of MytCc since it has recently been shown that the reduced forms of some HDPs, such as the the human β-defensin 1 [23,24], possess higher activity than the oxidized ones.

In contrast to that reported for oxidised isoforms of the Myt family peptide [25,26], our first analysis of the synthetic red-MytCc in solution and at neutral pH showed that the petide was not active against bacteria. Nonetheless, it was able to moderately inhibit the infectivity of an fish enveloped rhabdovius, VHSV (~40%) *in vitro*. Likewise, antiviral activity against a virus infecting cultured shrimps (white spot syndrome virus, WSSV) has been described for Mytilin [27], another HDP found in Mediterranean mussels. However, concentrations of Mytilin B needed to abolish the infectivity of WSSV are 2-fold lower than those of synthetic red-MytCc to partially inhibit VHSV infectivity. Intriguingly, synthetic red-MytCc immunomodulatory properties, such as chemotaxis, remain active in those experimental conditions.

Since the results related to antimicrobial activity and immunomodulatory properties of syntetic red-MytCc were partially contradictory, we addressed the question of whether or not the activity of this reduced HDP analogue could be environmentally sensitive. Having established that synthetic red-MytCc is an innate immunity effector at neutral pH and low concentration (from 0.1 to 1 μg/mL) such as it is expected from a chemokine-like molecule that is secreted outside of the cells, we focused our attention on determining the experimental conditions in which this HDP could act as a direct antimicrobial effector molecule.

Previous reports have shown that myticins as well as other mussel HDPs predominantly reside in cytoplasmic granules [26,28] of hemocytes, a phagocytic cell type in molluca [26]. Therefore, it seemed possible that the antimicrobial activity of MytCc could be directly exerted inside the granules after pathogen phagocytosis. This assumption is supported by the fact that viral replication could not be detected in the cells expressing recombinant Myt C but was evident in the surrounding non-expressing cells [10]. Moreover, it has been described that both phagocytic and non-phagocytic cells can internalize bacteria which then co-locate with granular antimicrobial peptides [29]. If this is the case, MytCc should act in environmental conditions at high concentrations and maybe low pH, since hemocytes contain eosinophilic granules [26].

As a first approach to evaluate this possibility and considering that antimicrobial peptides interact with cell membranes through electrostatic forces leading to their disorganization [30], we determined the ability of synthetic red-MytCc to promote aggregation of vesicles of different phospholipid composition at neutral and acid pH. As seen in most antimicrobial cationic peptide studies [3,31,32], red-MytCc interacted to a greater extent with anionic (PS) than with a zwitterionic phospholipids (PC) both at neutral and acidic pH. However, its ability to aggregate artificial vesicles of anionic phospholipids, and therefore, to perturb the membranes was three-fold higher at acidic pH. Similarly, at pH 5.5, clavanin A, an HDP expressed by the hemocytes of the ascidian *Styela clava*, induced membrane destabilisation and permeation more effectively than at physiological pH [33]. In parallel, infrared spectroscopic data indicated that the structure of synthetic red-MytCc is pH dependent, when decreasing the pH the content of alpha helix secondary structure increase and random structures were reduced. A similar structural change also accompanied by an increased antibacterial activity, in this case induced by the redox-regulation, has been reported for the human β-defensin 1 [34,35]. On the other hand, the fact that we could not observe thermal transitions of the red-MytCc at acidic pH, whereas this phenomenon was clearly seen at neutral pH (92–105 °C), indicates that the more structured form of the red-MytCc in acidic conditions corresponds with an extreme high thermal stability. Altogether, these results suggest that the increased capability of synthetic red-MytCc to interact and aggregate anionic phospolipids is attributable to its more structured state in an acidic environment. Furthermore, this combination of high percentage of α-helix and thermal stability probably made this molecule very resistant to the attack by proteases.

In accordance with biophysical data and taking into account that bacterial cell membranes are composed of a high proportion of acidic phospholipids which confer a negative charge to the surface [6,36], a dose-dependent antibacterial activity of red-MytCc at acidic pH was observed. A similar result of activity at low pH has been described for HDPs from other marine organisms [33,37].

Therefore, and although its definite *in vivo* relevance remains to be demonstrated, these findings provide further evidence that environmental factors strongly influence the direct antimicrobial activity of HDP and open new opportunities to design anti-infective that would function selectively in specific environments such as the skin, or the oral cavity, and that are sorely needed in this “antibiotic-resistance era”. In addition, the high thermal stability of red-MytCc prompted the use of this HDP as an alternative to chemical food preservatives.

## 4. Experimental Section

### 4.1. Reagents

Synthesis of Myticin C variant C peptide, (full length prepropeptide sequence, EMB CAM56832.1) red-MytCc [10] (QSVACRSYYCSKFCGSAGCSLYGCYLLHPGKICYCLHCSR, m.w. = 4433.26 Da, theoretical net charge +3 and +5 at pH 7 and 3, respectively accordingly to JaMBW software [38], was performed by Shine Gene (Shanghai, China) to a purity of >95%, as determined by high-performance liquid chromatography and mass spectrophotometry. Residual trifluoroacetic acid used both in the peptide synthesis and in the HPLC mobile phase (trifluoroacetate has a strong infrared absorbance at 1673 cm^−1^, which interferes with the characterization of the peptide amide I′ band) was removed by several lyophilization-solubilisation cycles in 10 mM HCl. Finally, red-MytCc was reconstituted to a final concentration of 1 mg/mL in Milli Q water and stored until used in suitable aliquots at −80°. Deuterium oxide (D_2_O, 99.9% by atom) was purchased from Sigma-Aldrich. The phospholipids phosphatidylcholine (PC) and phosphatidylserine (PS) (Avanti Polar Lipids) were derivatives of egg yolk and brain, respectively. All phospholipids were dissolved in chloroform and divided into aliquots, and the solvent was driven off under a stream of nitrogen and under vacuum. The resulting dry lipid films were suspended in the required D_2_O buffer at concentrations up to 3 mM in terms of lipid phosphorus [36], frequently vortexed and sonicated in a bath-type sonicator until the samples became completely transparent.

### 4.2. Cell Cultures and Virus

The fish cell line EPC [37] purchased from the American Type Culture Collection (ATCC number CRL-2872) was used in this work. Recently, the ATCC has revealed that the EPC cell line, originally deposited as a carp (*Cyprinus carpio*) cell line, is in fact a fathead minnow (*Pimephales promelas*) cell line. EPC cells were maintained at 28 °C in a 5% CO_2_ atmosphere with RPMI-1640 Dutch modified (Gibco, Invitrogen corporation, UK) cell culture medium containing 10% fetal calf serum (FCS) (Sigma, Che. Co., St. Louis, MO, USA), 1 mM Pyruvate (Gibco, Invitrogen corporation, UK), 2 mM Glutamine (Gibco), 50 μg/mL gentamicin (Gibco) and 2 μg/mL fungizone.

Viral haemorrhagic septicaemia virus strain 07.71 (VHSV_07.71_) isolated in France from rainbow trout (*Oncorhynchus mykiss*) [39] was propagated in EPC cells at 14 °C as previously reported [40]. Supernatants from VHSV_07.71_ infected EPC cell monolayers were clarified by centrifugation at 4000× *g* during 30 min and kept in aliquots at −70 °C. Clarified supernatants were used for the experiments. The virus stock was titrated in 96-well plates using a previously developed immunostaining focus assay (focus forming units, f.f.u.) [41,42].

### 4.3. Bactericidal Assay of Synthetic Myt C

To assay for synthetic red-MytCc antibacterial activity a previously developed protocol was used [7]. Briefly, 100 μL of an exponential-phase culture of *Escherichia coli* (*E.coli* Top F10 strain) were diluted in fresh LB medium, aliquots of 10 μL plated and the number of colony-forming units (CFU) per mL assayed in semisolid agar. Antibacterial activity of synthetic red-MytCc was determined by incubating ~10^3^ CFU of *E. coli* in 90 μL of LB media in the presence of different concentrations of synthetic red-MytCc (from 2.5 to 250 μM) diluted in water at pH 7 or 3 (final volume of 10 μL) in wells of 96-well plates. Controls consisted in bacterial culture incubated with 10 μl of sterile water or 14 mM Ampicillin (Sigma, Che. Co., St. Louis, MO, USA). Plates were incubated overnight at 37 °C and the next day the *E.coli* growth was determined colorimetrically using iodonitrotetrazolium violet dye (INT) (Sigma, Che. Co., St. Louis, MO, USA). For that, 50 μL of INT were added to each well to reach a final concentration of 0.25 mg/mL and incubated 30 min at 37 °C. Finally, the red coloured signal was measured at 570 nm using a spectrophotometer (SPECTROstar Omega, BMG Labtech GmbH, and Offenburg, Germany). Results were expressed as bacterial growth percentage and were calculated by the formula, (absorbance at 570 nm of *E. coli* in the presence of synthetic red-MytCc or ampicillin/absorbance at 570 nm of *E. coli* in the absence of synthetic red-MytCc or ampicillin) × 100.

### 4.4. *In Vitro* Cell Viability Assays

The cytotoxic effects of synthetic red-MytCc on EPC cell monolayers were determined by quantifying the EPC cell viability using an MTT (3-(4,5-Dimethyl-2-thiazolyl)-2,5-diphenyl-2*H*-tetrazolium bromide) Cell Titer 96, Non-Radioactive Cell Proliferation Assay (Promega, Mannheim, Germany). Cytotoxicity was examined following 3 days of EPC cell monolayers exposure to 6.25 to 125 μM of synthetic red-MytCc. The results were expressed as percentage of the control and calculated by the formula (absorbance at 570 nm of synthetic red-MytCc treated EPC cells/absorbance at 570 nm of untreated EPC cells) × 100.

### 4.5. Viral Infectivity Assays

To assay the influence of a synthetic antimicrobial peptide on VHSV infectivity, a previously developed immunostaining assay was used [43]. Briefly, to test the influence of pre-incubation of VHSV with red-MytCc, different concentrations of synthetic red-MytCc (up to 125 μM) was incubated with 10^3^ ffu from replication-competent stocks of concentrated VHSV (10^10^ ffu/mL) for 12 h at 14 °C in 25 μL serum-free cell culture medium supplemented with 2 mM l-glutamine and 50 μg/mL gentamicin. After incubation, VHSV-red-MytCc mixtures were added to the EPC cell monolayers, grown in 96 well plates, in a final volume of 100 μL per well. Alternatively, EPC cell monolayers were infected with VHSV (m.o.i. of 10^−3^) in the presence or absence of different concentrations of synthetic red-MytCc. In both cases, 2 h after infection at 14 °C, the cell monolayers were washed, 100 μL of fresh medium added to each well and plates further incubated for 24 h at 14 °C. The cell monolayers were then fixed for 10 min in cold methanol and air-dried. Monoclonal antibody (MAb) 2C9 directed towards the N protein of VHSV diluted 1000-fold in dilution buffer (0.24 mM merthiolate, 5 g/L Tween 20, 50 mg/L of phenol red in PBS pH 6.8) were added to the wells (100 μL/well) and incubated for 1 h at room temperature. After washing with distilled water, 100 μL of peroxidase-labelled rabbit anti-IgG mouse antibody (Ab) (Nordic, Tilburg, The Netherlands) were added per well, and incubation was continued for 30 min. After three washings by immersion in distilled water, 50 μL of 1 mg/mL per well of diaminobenzidine (DAB) (Sigma) in PBS containing H_2_O_2_ were added [44] and the reaction allowed to proceed until brown foci were detected with an inverted microscope (Nikon Eclipse TE2000-U, Nikon instruments Inc., Melville, NY, USA). Once washed with water and air dried, brown foci of DAB stained cells (VHSV-infected cell foci) were counted with an inverted microscope with a 10× ocular eye grid [41]. The results were expressed as the percentage of infectivity and calculated by the formula: (number of VHSV-infected cell foci in the presence of MytCc/total number of VHSV-infected cell foci in the absence of red-MytCc) × 100. Two independent experiments, each by triplicate, were performed.

### 4.6. Chemotaxis Assays

Chemotactic properties of the synthetic red-MytCc were determined using PET cell culture inserts of 8.0 mm pore size (Becton & Dickinson, Franklin Lakes, NJ, USA) in 24-well plates. Briefly, 250 μL of hemolymph from individual mussels (*n* = 8) was added to the upper compartment, and 400 μL of FSW (filtered sea water) or FSW containing red-MytCc at 0.25 or 0.025 μM were located in the lower compartment. As control, chambers containing 0.25 μM of red-MytCc in FSW in both upper and lower compartments were used. After 4 h of incubation in the dark at 15 °C, cells in the lower compartment were recovered, centrifuged for 750 rpm, 5 min in a cytocentrifuge (Cytospin 4 Cytocentrifuge, Thermo Scientific, Waltham, MA, USA) and stained using the Hemacolor kit (Merck, Darmstadt, Germany) according to the manufacturer’s instructions. Cells in the lower chamber were counted using a Nikon Eclipse 80i light microscope. The results were expressed as fold change of each treatment related to control ± SD. Pictures were taken using a Nikon Eclipse TS100 light microscope with a Nikon DS-Fi1 camera (Nikon Instruments Inc., Melville, NY, USA).

### 4.7. Lipid Vesicle Preparation

A phospholipid film was obtained upon overnight drying of a chloroform solution under vacuum. The phospholipids were suspended at a concentration of 1 mg/mL in medium buffer (10 mM sodium acetate, 10 mM 3-(*N*-morpholino)propionesulphonic acid, 10 mM 3-[cyclohexylamino]-1-propanesulphonic acid, 100 mM NaCl, 0.1 mM EDTA, and 0.01% NaN_3_; adjusted to a pH 3 and 7, respectively) for 1 h at 37 °C and vortexed vigorously. This suspension was subjected to 20 cycles of extrusion in a LoposoFast Basic extrusion apparatus with 100-nm polycarbonate filters (Avestin Inc., Mannheim, Germany). A 0.14 mM phospholipid final concentration was used in all the experiments.

### 4.8. Vesicle Aggregation Assay

Vesicle aggregation assays were carried out at previously described [45]. The optical density (OD) variation at 360 nm produced by the addition of the synthetic red-MytCc from the DMSO stock solution to a phospholipid vesicle suspension in medium buffer at the appropriate pH was measured on a Cary-Varian spectrophotometer after 1 h incubation at 37 °C. To account for the absorbance of phospholipid vesicles and peptide alone, control samples containing equal amounts of DMSO, but in the absence of peptide or phospholipid, were included.

### 4.9. FTIR Measurements

For amide I′ band recordings, lyophilized aliquots (300 μg) of the synthetic peptides and desired lipids (500 μg) were separately hydrated in 20 μL of D_2_O buffers (10 mM sodium acetate, 10 mM 3-(*N*-morpholino)propionesulphonic acid, 10 mM 3-[cyclohexylamino]-1-propanesulphonic acid, 100 mM NaCl, 0.1 mM EDTA, and 0.01% NaN_3_; adjusted to a pH 3 and 7, respectively; [46]) to avoid the interference of H_2_O infrared absorbance (1645 cm^−1^, [47]). The peptide or peptide-lipid solutions were mixed by placing them together in a liquid demountable cell (Harrick, Ossining, NY, USA) equipped with CaF_2_ windows and 25-μm-thick mylar spacers, and maintained at room temperature for ~30 min to ensure that the isotopic H-D amide proton exchange reached equilibrium, as judged by a constant minimum absorbance at the residual amide II band (1547 cm^−1^). The peptide concentration in the final buffers solution was 3.4 mM and the same for the lipid-peptide mixtures; and the phospholipids were 32.5 mM. The signal:noise ratio of the spectra was better than 10,000:1. Buffer contributions were subtracted, and the resulting spectra were used for analysis. Band decomposition of the original amide I′ has been described previously [18]. For temperature-dependent studies, the samples were submitted to heating cycles at each at the indicated temperatures. Each step in such heating cycles include (i) a step-like increase in temperature, (ii) an stabilization period of the sample (or plain buffer) in the infrared cell at each temperature and (iii) a period of spectral acquisition. The duration of a complete heating cycle was of approximately 3 h. Protein thermal melting was monitored by FTIR by following the changes induced in the width at half height of the amide I′ band by temperature. The thermal midpoint, *T*_m_, was determined by using a two-state folding mechanism [18].

### 4.10. Sigmoidal Data Fitting Analysis

Certain infrared and dichroism data presenting a sigmoid behavior cannot be satisfactorily explained with the typical Boltzmann equation, especially if they have big negative or positive sloping bottom and top plateau. From the Boltzmann equation we have developed a new equation that takes into account the positive or negative plateau.

Boltzmann:

*y* = *A*2 + (*A*1 − *A*2)/(1 + exp((*x* − *x*_0_)/d*x*)) (1)

Boltzmann Modiﬁed:

*y* = *A*_2_ + *B*_2_ × *x* + (*A*_1_ + *B*_1_ × *x* − *A*_2_ − *B*_2_ × *x*)/(1 + exp((*x* − *x*_0_)/d*x*)) (2)

where *A*_1_ is the initial value (left horizontal asymptote), *B*_1_ is the slope for the initial asymptote, *A*_2_ is the final value (right horizontal asymptote), *B*_2_ is the slope for the final asymptote, *x*_0_ is the center (point of inflection), and d*x* is the width (the change in *x* corresponding to the most significant change in *Y* values). Curve fitting analysis using “Boltzmann Modiﬁed” equation was performed with OriginLab 7.0 program.

### 4.11. Statistical Analysis

Statistical comparisons were made using a one-tailed Student *t*-test considering groups of equal variance. Differences were considered statistically significant with *p* ≤ 0.01 or *p* ≤ 0.05.

## 5. Conclusions

The data shown in this work highlight the dynamic and environmentally sensitive behavior of the mussel reduced HDP analogue MytCc (re-MytCc) and suggest that its direct antimicrobial activity is exerted at acid pH and high concentration, may be the form in which this peptide is acumulated in mussel phagocytic cells. In contrast, its immunomodulatory properties, as the chemotactic activity, are shown at neutral pH and low concentration. Overall, our results depict Myt C as a potential new and versatile marine-peptide template for the development of bioactive products with application in different fields and environmental conditions.
